# Protective effects of aerobic exercise on acute lung injury induced by LPS in mice

**DOI:** 10.1186/cc11807

**Published:** 2012-10-18

**Authors:** Cintia Tokio Reis Gonçalves, Carlos Gustavo Reis Gonçalves, Francine Maria de Almeida, Fernanda Degobi Tenório Quirino dos Santos Lopes, Ana Carolina Cardoso dos Santos Durão, Fabiana Almeida dos Santos, Luiz Fernando Ferraz da Silva, Tania Marcourakis, Hugo C Castro-Faria-Neto, Rodolfo de Paula Vieira, Marisa Dolhnikoff

**Affiliations:** 1 Departamento de Patologia (LIM05) da Faculdade de Medicina da Universidade de São Paulo, Av. Dr. Arnaldo, 455 sala 1155, CEP 01246-903, São Paulo, Brazil; 2 Departamento de Clínica Médica (LIM20) da Faculdade de Medicina da Universidade de São Paulo, Av. Dr. Arnaldo, 455 sala 1226, CEP 01246-903, São Paulo, Brazil; 3 Laboratório de Imunofarmacologia, Instituto Oswaldo Cruz, Fiocruz, Av. Brasil, 4365, CEP 21045-900, Rio de Janeiro, Brazil; 4 Departamento de Análises Clínicas e Toxicológicas, Faculdade de Ciências Farmacêuticas, Universidade de São Paulo, Av. Prof. Lineu Prestes, 580 Bl 13B, CEP 05503-900, São Paulo, Brazil; 5 Universidade Nove de Julho - UNINOVE, Rua Vergueiro 239/245, Vergueiro, CEP 01504-000, São Paulo - SP, Brazil

## Abstract

**Introduction:**

The regular practice of physical exercise has been associated with beneficial effects on various pulmonary conditions. We investigated the mechanisms involved in the protective effect of exercise in a model of lipopolysaccharide (LPS)-induced acute lung injury (ALI).

**Methods:**

Mice were divided into four groups: Control (CTR), Exercise (Exe), LPS, and Exercise + LPS (Exe + LPS). Exercised mice were trained using low intensity daily exercise for five weeks. LPS and Exe + LPS mice received 200 µg of LPS intratracheally 48 hours after the last physical test. We measured exhaled nitric oxide (eNO); respiratory mechanics; neutrophil density in lung tissue; protein leakage; bronchoalveolar lavage fluid (BALF) cell counts; cytokine levels in BALF, plasma and lung tissue; antioxidant activity in lung tissue; and tissue expression of glucocorticoid receptors (Gre).

**Results:**

LPS instillation resulted in increased eNO, neutrophils in BALF and tissue, pulmonary resistance and elastance, protein leakage, TNF-alpha in lung tissue, plasma levels of IL-6 and IL-10, and IL-1beta, IL-6 and KC levels in BALF compared to CTR (*P *≤0.02). Aerobic exercise resulted in decreases in eNO levels, neutrophil density and TNF-alpha expression in lung tissue, pulmonary resistance and elastance, and increased the levels of IL-6, IL-10, superoxide dismutase (SOD-2) and Gre in lung tissue and IL-1beta in BALF compared to the LPS group (*P *≤0.04).

**Conclusions:**

Aerobic exercise plays important roles in protecting the lungs from the inflammatory effects of LPS-induced ALI. The effects of exercise are mainly mediated by the expression of anti-inflammatory cytokines and antioxidants, suggesting that exercise can modulate the inflammatory-anti-inflammatory and the oxidative-antioxidative balance in the early phase of ALI.

## Introduction

Acute lung injury (ALI) and its most severe presentation, acute respiratory distress syndrome (ARDS), are clinical disorders characterized by hypoxemic respiratory failure associated with acute pulmonary inflammation secondary to several different etiologies and exhibit high mortality rates [1].

The regular practice of exercise has been increasingly associated with beneficial effects on chronic pulmonary conditions such as asthma and chronic obstructive pulmonary disease [2,3]. However, few studies have investigated the effects of aerobic exercise on acute lung injury (ALI). In 2008, Mussi *et al. *demonstrated the beneficial effects of exercise in a rat model of pulmonary inflammation induced by lung ischemia-reperfusion [4]. Some effects of exercise have been reported also in lipopolysaccharide (LPS) models of ALI. Cannon and Kluger showed in 1984 that mice trained on running wheels and then injected with *Salmonella typhimurium *had a significantly higher survival rate than sedentary mice [5]. More recently, Ramos *et al. *showed that exercise reduced neutrophilic inflammation and exhaled NO levels in mice submitted to intra-nasal instillation of LPS [6]. LPS is one of the major proinflammatory constituents of the cell walls of gram-negative bacteria and has been largely used in animal models of acute lung inflammation where neutrophils, acute phase cytokines and reactive oxygen species play major roles [7].

In this study, we investigated the mechanisms involved in the protective effect of exercise in a model of LPS-induced ALI. For this purpose, we analyzed lung mechanics, lung tissue inflammation, protein leakage, pulmonary and systemic cytokine levels, the expression of glucocorticoid receptor (Gre) in lung tissue and pulmonary antioxidant enzyme activity in mice instilled with LPS after five weeks of daily aerobic exercise.

## Materials and methods

This study was approved by the review board for human and animal studies of the School of Medicine at the University of São Paulo (São Paulo, Brazil), protocol 0402/08. All animals in the study received humane care in compliance with the Guide for the Care and Use of Laboratory Animals (NIH publication 85-23, revised 1985).

### Animals and experimental design

Thirty-two male BALB/c mice (20g to 25 g) were divided into four groups (n = 8 in each group): Control (non-exercised and non-LPS-instilled), Exercise (Exe; exercised and non-LPS-instilled), LPS (non-exercised and LPS-instilled), Exe + LPS (exercised and LPS-instilled).

### Aerobic exercise treadmill test and exercise conditioning

Animals were initially adapted to the treadmill for three days (15 minutes, 25% inclination, 0.2 km/hour). Afterwards, a maximal exercise capacity test (test 1) was performed with a 5-minute warm-up (25% inclination, 0.2 km/hour) followed by an increase in treadmill speed (0.1 km/hour every 2.5 minutes) until animal exhaustion, that is, until they were not able to run even after 10 mechanical stimuli. Maximal aerobic capacity (100%) was established as the maximal speed was reached by each animal. Mice from the Exe and Exe + LPS groups were then trained using low intensity exercise (50% of the mean maximal speed) for 60 minutes/day, 3 days/week, for 5 weeks [3]. The maximal exercise capacity test was repeated for all animals after five weeks (test 2). The exercise protocol is illustrated in Additional files 1, 2 and 3.

### LPS instillation

Forty-eight hours after the last exercise capacity test, animals from the LPS and Exe + LPS groups received one intratracheal instillation of 200 µg of *Escherichia coli *LPS (L3755, Sigma Aldrich, St. Louis, MO, USA) suspended in saline solution (total volume = 0.05 ml per animal). Animals from the Control and Exe groups received one intratracheal instillation of 0.05 ml saline solution. For intratracheal instillation, the mice were anesthetized with ketamine (0.04 ml/mice) and xylasine (0.1 ml/mice); then, a 1 cm-long midline cervical incision was made to expose the trachea, and LPS or saline was instilled using a bent 27-gauge tuberculin needle. The cervical incision was closed with a 5.0 silk suture, and the mice were returned to their cages. The animals recovered rapidly after surgery [8].

### Measurement of the exhaled nitric oxide (eNO) concentration

Twenty-four hours after the intratracheal instillation with saline or LPS, the animals were anesthetized using pentobarbital sodium (50 mg/kg i.p.), tracheostomized, and mechanically ventilated at 60 breaths/min with a tidal volume of 10 ml/kg using a Harvard 683 ventilator (Harvard Apparatus, South Natick, MA, USA). eNO levels were measured at the expiratory port of the ventilator using a Mylar bag (3 to 6) for five minutes. The concentrations of eNO were measured by chemiluminescence using a fast-responding analyzer (NOA 280; Sievers Instruments Inc., Boulder, CO, USA). Before each measurement, the analyzer was calibrated with a certified 47-ppb NO source (White Martins, São Paulo, Brazil) and a zero NO filter (Sievers Instruments Inc.). To avoid environmental contamination, an NO filter was attached to the breathing circuit [9].

### Respiratory system mechanics

The tracheal pressure (Ptr) was measured using a pressure transducer (DP 45-28-2114, Validyne, Northridge, CA, USA) connected to a side tap in the orotracheal cannula. Airflow (V^. ^) was measured using a pneumotachograph (Fleisch-4.0, OEM Medical, Richmond, VA, USA) attached to the tracheal cannula and to a differential pressure transducer (Validyne DP 45-16-2114). Ptr and V^. ^signs were registered using a Gould RS-3400 recorder (Gould Instruments, Cleveland, OH, USA), sampled at 200 Hz using an analog-to-digital converter (DT 2801 A, Data Translation, Marlboro, MA, USA), and stored in a microcomputer. Lung volume (V) changes were obtained by electronic integration of V^. ^. The respiratory system resistance (Rrs) and elastance (Ers) were computed by least squares fitting of the measured values of Ptr, V, and V^. ^over 9 to 10 respiratory cycles using the following equation of motion of the respiratory system:

Ptr (t) = Ers × V(t) + Rrs × V^. ^(t), where t is time. All data were collected and processed using LABDAT software (RHT-InfoData, Montreal, Quebec, Canada) [10].

### Total and differential cell counts in bronchoalveolar lavage fluid (BALF)

After the mechanical parameters were measured, the mice were euthanized by exsanguination (blood collection through the abdominal vein), and BALF was collected by flushing the lungs three times with 0.5 ml of 37°C sterile, pyrogen-free, physiological saline (0.9% NaCl) via the tracheal cannula. After BALF collection, the samples were centrifuged at 420 × *g *at 4°C for 15 minutes, and the supernatant was stored at -70°C for subsequent analysis of protein and cytokine levels. The cell pellets were resuspended in 1 ml of PBS, and the total cell counts were performed using a Neubauer chamber. For differential cells counts, cytospin slides were prepared and stained with Diff-Quick; 300 cells were counted per slide [3,11].

### Evaluation of cytokines in BALF

Levels of IL-1β, IL-6, KC (the murine functional homolog to IL-8), IL-10 and TNF-α in the cell-free BALF were evaluated using ELISA in accordance with the manufacturer's instructions (Duo Set, R&D Systems, Minneapolis, MN, US) [12].

### Protein leakage in BALF

Proteins from lung lavage supernatant were quantified using the bicinchoninic acid (BCA) method to evaluate lung vascular permeability [11].

### Evaluation of plasma cytokines

Blood was collected through the abdominal vein for the measurement of cytokines. The blood was put on ice, and plasma was collected by centrifugation at 800 × *g *for 15 minutes at 4°C, aliquoted and stored at -70°C until analyzed. Levels of IL-6 and IL-10 were evaluated using ELISA in accordance with the manufacturer's instructions (Duo Set, R&D Systems, Minneapolis, MN, USA) [12].

### Lung morphometry

Lung tissue was fixed in buffered 10% formaldehyde for 24 hours, routinely processed and embedded in paraffin. Five-µm-thick slides were stained with H & E for analysis of the polymorphonuclear (PMN) cell content [13]. The expression of IL-6, IL-10, TNF-α, SOD-2, and glucocorticoid receptor was assessed with immunohistochemistry as previously described [3]. Briefly, sections were deparaffinized and 0.3% hydrogen peroxide was applied for 35 minutes to inhibit endogenous peroxidase activity. Antigen retrieval was performed using citrate solution for 45 minutes to inhibit endogenous peroxidase activity. The sections were incubated overnight at 4°C with anti-IL-6 (1:200) (R&D Systems, Minneapolis, MN, USA), anti-IL10 (1:900), anti-TNF-α (1:400), anti-SOD-2 (1:400) and anti-Gre (1:500) (Santa Cruz Biotechnology Inc., Santa Cruz, CA, USA). An ABC Vectstain Kit (Vector Elite PK-6105, Vector Laboratories, CA, USA) was used as the secondary antibody, and 3,3 diaminobenzidine (Sigma) was used as the chromogen. The sections were counterstained with Harris hematoxylin (Merck, Darmstadt, Germany).

Using conventional morphometry, we assessed the density of PMN cells and cells that were positive for IL-6, IL-10, TNF-α, SOD-2 and Gre within the alveolar parenchyma. Using a 100-point grid of known area (62,500 µm^2 ^at × 400 magnification) attached to the ocular of the microscope, we counted the number of points hitting alveolar tissue and the number of PMN and positive cells in each field. The cell density was determined as the number of positive cells in each field divided by the tissue area and expressed as cells/mm^2 ^[13]. The morphometric measurements were performed on 15 fields for each animal at x400 magnification by an investigator blinded to the group studied.

### Antioxidant enzyme activity assays

The enzymatic activity of catalase (CAT) was determined in lung homogenates using spectrophotometry. Catalase activity was evaluated by measuring the consumption of hydrogen peroxide according to Aebi [14]. The decrease in absorbance was monitored spectrophotometrically (Biochrom Libra S12) at 25°C and 240 nm for 60 seconds.

Glutathione peroxidase (GPX) activity was determined using tert-butylhydroperoxide as substrate, and the formation of oxidized glutathione (GSSG) was indirectly monitored spectrophotometrically through NADPH consumption detected for five minutes at 340 nm [15].

Glutathione reductase (GR) activity was determined based on the method of Carlberg and Mannervik [16]. The reduction of GSSG to GSH was measured through NADPH consumption, which was monitored spectrophotometrically at 37°C and 340 nm for five minutes.

Glutathione S-transferase (GST) activity was determined by measuring the conjugation of 1-chloro-2,4- dinitrobenzene (CDNB) with reduced glutathione based on the method of Habig *et al. *[17]. The formation of the complex was monitored spectrophotometrically at 25°C and 340 nm for five minutes. The GPX, GR and GST assays were performed using a Power Wave × 340 spectrophotometer (Bio-Tek Instruments Inc., software KC4 v3.0).

### Malondialdehyde (MDA)

MDA was quantified in homogenates of lung tissue precipitated with 7.2% trichloroacetic acid solution, centrifuged, and incubated with thiobarbituric acid (TBA) at 0.6% (Sigma) for 45 minutes at 90°C [18]. Then, the supernatant was cooled for five minutes, and n-butanol was added to extract the color product of the aqueous solution. After centrifugation, the MDA in the butanol layer was measured at 532 nm. HPLC analysis was performed using a LC-VP HPLC system from Shimadzu (Kyoto, Japan). The concentration of total protein in each sample of homogenate was determined using the Bradford Method and BSA as a standard (1 mg/ml). MDA was expressed in nanomoles per milligram of total protein.

### Statistical analysis

Statistical analysis was performed using the SPSS 15.0^® ^software package (SPSS, Chicago, Illinois, USA). The normality of the data was tested using the Kolmogorov-Smirnov test. Logarithmic data transformation was applied when the data followed a nonparametric distribution. Pulmonary resistance was the only parameter that showed a parametric distribution after logarithmic transformation. A two-way analysis of variance was performed to resolve the main effects of LPS and exercise on the parameters analyzed. This test also produces an interaction term that identifies whether the effects of one factor (LPS) are influenced by the effects of the other (exercise). The associations among the various parameters in LPS-instilled animals were evaluated using Pearson or Spearman tests, depending on data distribution. The results are expressed as the mean ± standard deviation or median (interquartile range). The level of significance was set at *P *<0.05.

## Results

### Aerobic exercise treadmill test and exercise conditioning

Non-trained animals showed a significant decrease in maximal exercise capacity between the first and second tests (test 1 = 1.5 km/hour, test 2 = 1.3 km/hour, *P*= 0.001). Trained animals showed no difference in maximal exercise capacity between the first and second tests (test 1 = 1.6 km/hour, test 2 = 1.6 km/hour).

### Respiratory mechanics

**Figure 1:**
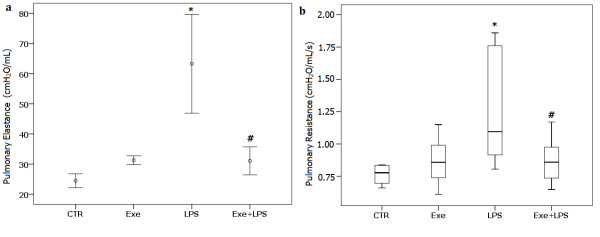
**Values of pulmonary elastance (a) and pulmonary resistance (b)**. * indicates significant effect of LPS in increasing pulmonary resistance and elastance (*P *= 0.03). # indicates significant effect of exercise in attenuating the effect of LPS on pulmonary resistance and elastance (*P *= 0.03). Exe (exercise): instilled with saline and trained; LPS: instilled with LPS and untrained; Exe+LPS: instilled with LPS and trained. LPS, lipopolysaccharide.

The graphs in Figure 1 show the pulmonary elastance and resistance in the four groups. There was a significant effect of LPS in increasing both pulmonary resistance and elastance (*P *= 0.03). We observed an effect of exercise in attenuating the effect of LPS on pulmonary resistance and elastance (*P *= 0.03).

### Concentration of exhaled NO

**Figure 2:**
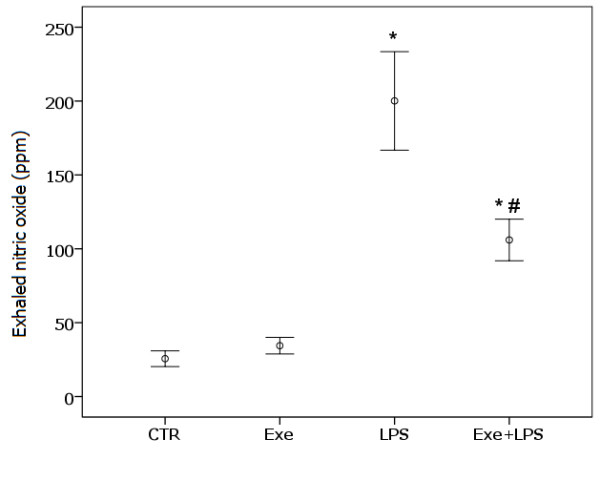
**Values of exhaled nitric oxide**. * indicates significant effect of LPS in increasing exhaled nitric oxide (*P *<0.001). # indicates significant effect of exercise in attenuating the effect of LPS on exhaled nitric oxide (*P *= 0.01). CTR (control): instilled with saline and untrained; Exe (exercise): instilled with saline and trained; LPS: instilled with LPS and untrained; Exe+LPS: instilled with LPS and trained. LPS, lipopolysaccharide.

Figure 2 shows the concentration of eNO in the four groups. LPS instillation determined an increase in the concentration of eNO (*P *<0.001). There was a significant effect of exercise in attenuating the effect of LPS on eNO (*P *= 0.01).

### Protein leakage in BALF

We observed an effect of LPS in increasing the protein concentration in BALF (*P *<0.001) but exercise did not attenuate this effect. CTR = 1.88 (0.59) µg/ml, Exe = 1.73 (0.43) µg/ml, LPS = 3.86 (1.99) µg/ml, and Exe + LPS = 3.41 (2.47) µg/ml.

### Cell counts in BALF

The cell counts in BALF are shown in Table 1. There was an effect of LPS in increasing the number of total cells and neutrophils in BALF (*P *<0.001); exercise did not attenuate this effect. There was no effect of LPS or exercise on the number of lymphocytes, macrophages and eosinophils in BALF.

**Table 1:** Total and differential cell counts in bronchoalveolar lavage fluid (cells/ml).

	Control	Exercise	LPS	Exercise+LPS	*P*
total cells	1.35 ± 1.71	1.30 ± 2.01	8.73 ± 3.08^a ^	7.30 ± 0.68	<0.001
neutrophils	0.04 ± 0.04	0.04 ± 0.05	7.28 ± 1.56^a ^	6.00 ± 3.02	<0.001
lymphocytes	0.04 ± 0.07	0.02 ± 0.02	0.15 ± 0.14	0.16 ± 0.20	ns
macrophages	1.11 ± 0.50	1.00 ± 0.60	1.12 ± 0.60	0.86 ± 0.60	ns
eosinophils	0.02 ± 0.04	0	0.01 ± 0.03	0.04 ± 0.05	ns

^a ^indicates the significant effect of LPS in increasing cell counts; values are expressed as the means ± SD. LPS, lipopolysaccharide; ns, non-significant.

### Plasma and BALF cytokine levels

Plasma and BALF cytokine levels are shown in Table 2. We observed an effect of LPS in increasing the plasma level of IL-6 (*P *<0.001) and IL-10 (*P *= 0.027), but no effect of exercise. There was an effect of LPS in increasing the levels of IL-6, IL-1β and KC in BALF (*P *≤0.005). Moreover, the results showed an interaction effect of LPS and exercise in increasing the IL-1β level in BALF (*P *= 0.04). There was no effect of LPS or exercise on the levels of TNF-α and IL-10 in BALF.

**Table 2:** Cytokine levels in plasma and BALF (ng/ml).

	Control	Exercise	LPS	Exercise+LPS	*P*
IL-6 BALF	0.015 ± 0.002	0.011 ± 0.005	0.541 ± 0.445^a ^	0.767 ± 0.664	<0.001
IL-6 plasma	0.016 ± 0.008	0.011 ± 0.003	0.096 ± 0.029^a ^	0.059 ± 0.037	≤0.001
IL-10 BALF	0.009 ± 0.032	0.050 ± 0.048	0.066 ± 0.072	0.056 ± 0.041	ns
IL-10 plasma	0.013 ± 0.034	0.058 ± 0.278	0.351 ± 0.176^a ^	0.111 ± 0.215	0.027
IL-1β BALF	0.150 ± 0.033	0.106 ± 0.060	0.161 ± 0.186^a ^	0.357 ± 0.246^b ^	<0.02
KC BALF	0.017 ± 0.011	0.013 ± 0.007	0.596 ± 0.233^a ^	0.626 ± 0.121	<0.001
TNF-α BALF	0.018 ± 0.016	0.028 ± 0.018	0.027 ± 0.017	0.032 ± 0.029	ns

^a ^indicates the significant effect of LPS in increasing cytokine levels; ^b ^indicates the significant interaction effect of LPS and exercise in increasing the cytokine level; values are means ± SD. BALF, bronchoalveolar lavage fluid; LPS, lipopolysaccharide; ns, non-significant.

### PMN cell content in lung tissue

**Figure 3:**
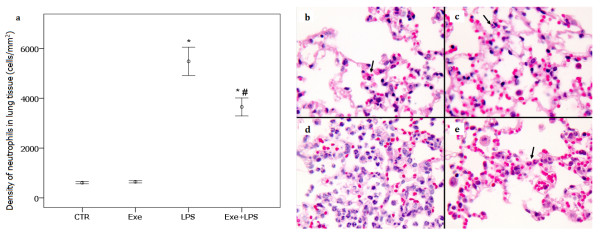
**Density of neutrophils in lung tissue**. In (**a**) * indicates significant effect of LPS in increasing the PMN cell content in lung tissue (*P *<0.001). # indicates significant effect of exercise in attenuating the effect of LPS on PMN cell content in lung tissue (*P *= 0.01). CTR (control): instilled with saline and untrained; Exe (exercise): instilled with saline and trained; LPS: instilled with LPS and untrained; Exe+LPS: instilled with LPS and trained. The microphotograph illustrates the density of neutrophils in the four groups: **b **= CTR, **c **= Exe, **d **= LPS, **e **= Exe+LPS. LPS, lipopolysaccharide; PMN, polymorphonuclear.

Figure 3 shows the PMN cell contents in lung tissue. LPS instillation resulted in an increase in the PMN cell content in lung tissue (*P *<0.001). There was a significant effect of exercise in attenuating the effect of LPS on PMN cell content (*P *= 0.01).

### Cytokine expression in lung tissue

**Figure 4:**
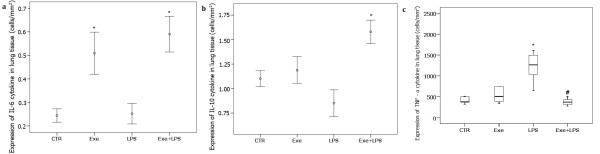
**Expression of IL-6 (a), IL-10 (b) and TNF-α (c) in lung tissue**. In (a) and (b) * indicates a significant effect of exercise in increasing the expression of IL-6 and IL-10 in lung tissue (*P *<0.001) and (*P *= 0.002), respectively. In (c) * indicates significant effect of LPS in increasing the expression of TNF-α in lung tissue (*P *= 0.001). # indicates significant effect of exercise in attenuating the effect of LPS on the expression of TNF-α in lung tissue (*P *= 0.001). CTR (control): instilled with saline and untrained; Exe (exercise): instilled with saline and trained; LPS: instilled with LPS and untrained; Exe+LPS: instilled with LPS and trained. LPS, lipopolysaccharide.

Figure 4 shows the expression of IL-6, IL-10 and TNF-α in lung tissue. There was no effect of LPS on the expression of IL-6 in lung tissue, but exercise induced an increase in IL-6 expression (*P *<0.001). In addition to a significant effect of exercise on IL-10 expression in lung tissue (*P *= 0.002), we observed a significant interaction effect of LPS and exercise on the expression of IL-10 (*P *= 0.013). LPS instillation resulted in an increase in the expression of TNF-α in lung tissue (*P *= 0.001). There was a significant effect of exercise in attenuating the effect of LPS on TNF-α expression in lung tissue (*P *= 0.001).

### Antioxidant enzyme activity and MDA levels in lung tissue

**Figure 5:**
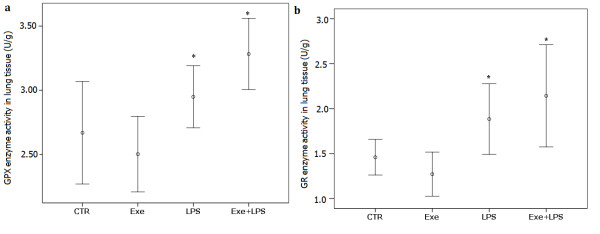
**GPX (a) and GR (b) enzymes activities in lung tissue**. * indicates significant effect of LPS in increasing the levels of GPX and GR (*P *<0.001). CTR (control): instilled with saline and untrained; Exe (exercise): instilled with saline and trained; LPS: instilled with LPS and untrained; Exe+LPS: instilled with LPS and trained. GPX, glutathione peroxidase; GR, glutathione reductase; LPS, lipopolysaccharide.

The antioxidant enzyme activity in lung tissue is shown in Figure 5. LPS instillation determined an increase in the levels of GPX and GR (*P *<0.001), but there was no effect of exercise. There was no effect of LPS or exercise on the levels of CAT, GST or MDA.

### SOD-2 expression in lung tissue

**Figure 6:**
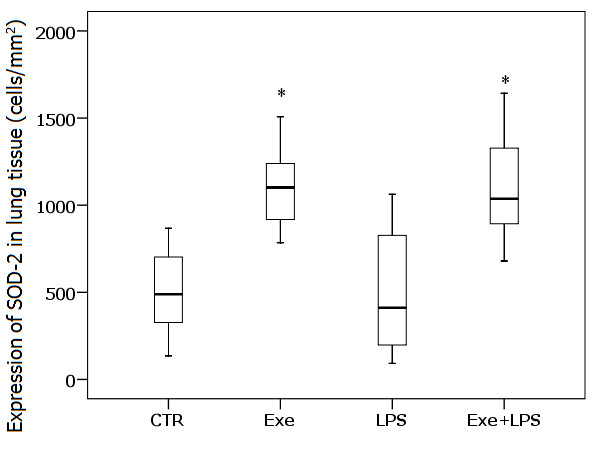
**SOD-2 expression in lung tissue**. * indicates significant effect of Exe in increasing the expression of SOD-2 in lung tissue (*P *<0.001). CTR (control): instilled with saline and untrained; Exe (exercise): instilled with saline and trained; LPS: instilled with LPS and untrained; Exe+LPS: instilled with LPS and trained. LPS, lipopolysaccharide; SOD-2 superoxide dismutase.

Figure 6 shows the levels of SOD-2 expression in lung tissue. The results demonstrated a significant effect of exercise on increasing SOD-2 expression in lung tissue (*P *<0.001) and no effect of LPS.

### Glucocorticoid receptor expression in lung tissue

**Figure 7:**
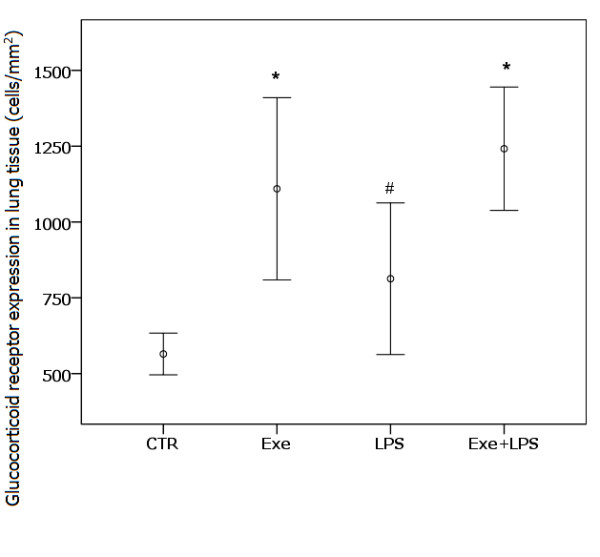
**Gre expression in lung tissue**. * indicates significant effect of exercise in increasing the expression of Gre in lung tissue (*P *<0.001). # indicates the marginal effect of LPS in this increasing (*P *= 0.05). CTR (control): instilled with saline and untrained; Exe (exercise): instilled with saline and trained; LPS: instilled with LPS and untrained; Exe+LPS: instilled with LPS and trained. Gre, glucocorticoid receptor; LPS, lipopolysaccharide.

Figure 7 shows the expression of Gre in lung tissue in the four groups. We observed a marginal effect of LPS (*P *= 0.05) and a significant effect of exercise (*P *<0.001) in increasing Gre expression. There was no interaction effect of LPS and exercise.

### Correlations

Table 3 lists the correlations among the levels of cytokines, inflammatory cell counts, eNO and mechanical parameters in animals that received a LPS instillation (LPS and Exe + LPS groups). We observed significant correlations among the levels of cytokines within the three studied compartments (BAL, plasma and lung tissue). There were also significant correlations between the density of neutrophils and the levels of IL-6 (R = - 0.64, *P *= 0.007), IL-10 (R = - 0.55, *P *= 0.02) and TNF-α (R = 0.58, *P *= 0.02) in lung tissue. Further, the levels of eNO were significantly correlated with values of pulmonary resistance (R = 0.73, *P *= 0.001) and elastance (R = 0.72, *P *= 0.002).

**Table 3:** Significant correlations between cytokine levels, inflammatory cell counts, exhaled NO, and mechanical parameters in the LPS and exercise + LPS groups.

	Neutro T	Neutro B	IL-10 T	IL-10 B	IL-6 T	IL-6 B	IL-1β B	Exhaled NO
Neutro T			R = -0.553 *P *= 0.026					
IL-10 P				R = 0.678 *P *= 0.015				
IL-6 T	R = -0.640 *P *= 0.007	R = -0.574 *P *= 0.020	R = 0.524 *P *= 0.037					
IL-6 B							R = 0.869 *P *< 0.001	
IL-6 P				R = 0.636 *P *= 0.026				
KC B				R = 0.504 *P *= 0.047		R = 0.729 *P *= 0.001	R = 0.653 *P *< 0.006	
TNF-α T	R = 0.578 *P *= 0.019		R = -0.685 *P *= 0.003		R = -0.597 *P *= 0.015			
Elastance								R = 0.718 *P *= 0.002
Resistance								R = 0.729 *P *= 0.001

BALF, bronchoalveolar lavage fluid; IL-1β B, IL-1β in BALF; IL-6 B, IL-6 in BALF; IL-6 S, IL-6 in plasma; IL-6 T, IL-6 in lung tissue; IL-10 B, IL-10 in BALF; IL-10 S, IL-10 in plasma; IL-10 T, IL-10 in lung tissue; KC B, KC in BALF; LPS, lipopolysaccharide; Neutro B, neutrophil in BALF; Neutro T, neutrophil in lung tissue; TNF-α T, TNF-α in lung tissue.

## Discussion

This study investigated the mechanisms involved in the protective effects of aerobic exercise in a model of ALI induced by LPS. LPS instillation resulted in increased levels of eNO, higher numbers of total cells and neutrophils in BALF, higher number of neutrophils and TNF-α positive cells in the lung parenchyma, higher values of pulmonary resistance and elastance, increased protein leakage, increases of IL-6 and IL-10 levels in plasma, increases of IL-1β, IL-6 and KC levels in BALF, and higher activities of GPX and GR in lung tissue. We demonstrated that the low intensity exercise performed during the five weeks before intratracheal instillation of LPS decreased pulmonary resistance and elastance, inhibited neutrophilic tissue infiltration and the expression of TNF-α in lung tissue, decreased the concentration of eNO, increased the expression of SOD-2, glucocorticoid receptors, IL-6 and IL-10 in lung tissue and the levels of IL-1β in BALF. These results suggest that aerobic exercise may play an important role in protecting the lung from the inflammatory effects of LPS-induced ALI by altering the inflammatory profile and increasing the expression of anti-oxidants in lung tissue.

Few studies to date have investigated the effects of aerobic preconditioning on the inflammatory changes involved in acute lung injury. MUSSI *et al. *evaluated the benefits of exercise in a rat model of pulmonary inflammation induced by lung ischemia-reperfusion and observed decreases in protein leakage and plasma levels of IL-1β and TNF-α and an increase in SOD activity in trained animals [4]. In the present study, we used intratracheal instillation of LPS as a model of ALI induced by gram-negative bacteria. The main difference between the study of MUSSI *et al. *and ours was the marked effect of exercise on decreasing LPS-induced pulmonary inflammation as assessed by neutrophilic infiltration and eNO.

We observed an effect of both LPS and exercise on the release of IL-6. IL-6 is one of the first acute phase cytokines released in sepsis/endotoxemia and is followed by increases in the levels of IL-1β, IL-8, TNF-α and IL-10 [2]. Consistently, there was an increase in IL-6 levels in both plasma and BALF of LPS-instilled animals in our model. Whether the increasing of pulmonary and plasmatic IL-6 in ALI is detrimental or protective is controversial. IL-6 was previously considered exclusively as a pro-inflammatory cytokine, and the circulating levels of IL-6 were directly correlated with the severity of ALI [19]. Currently, the anti-inflammatory effects of IL-6 have been demonstrated [20-24]. Evidence for the protective anti-inflammatory effects of IL-6 is derived from studies that show that endogenous IL-6 in endotoxin-induced ALI controls the level of pro-inflammatory cytokines and neutrophilic inflammation [24]; also, the administration of IL-6 induces SOD production and reduces LPS-induced neutrophilic infiltration in liver in a model of endotoxemia in rats [21]. Further, it has been reported that IL-6 plays a protective role in limiting the disruption of the alveolar barrier by neutrophils [23].

An exercise-induced increase in IL-6 expression in lung tissue was observed in our trained animals of both the Exe and LPS + Exe groups. Evidence regarding the effects of exercise on the release of IL-6 is accumulating [2,25,26]. An increase in circulating levels of IL-6 is consistently found during aerobic exercise; however, a decline in the levels of IL-6 has been observed during the post-exercise period [2,25]. The dynamics of the plasma variation of IL-6 levels induced by exercise could explain the low levels of plasmatic IL-6 observed in our Exe group because this analysis was performed 72 hours after the last training session.

IL-6 has been shown to stimulate the production of IL-10, a cytokine with well known anti-inflammatory effects [2,21,27,28]. Interestingly, in our animals the higher tissue expression of IL-6 was associated with the higher expression of IL-10 in lung tissue; we further observed a negative association between these two anti-inflammatory cytokines and the density of neutrophils in lung tissue.

Distinct mechanisms have been proposed for the anti-inflammatory effects of IL-6 in acute or chronic conditions [24]. In acute inflammatory responses, as those elicited by LPS, IL-6 operates to control the extent of tissue inflammatory responses by inhibition of pro-inflammatory cytokines and induction of anti-inflammatory molecules. When chronically released, as when induced by repeated bouts of exercise, IL-6 could induce the beneficial anti-inflammatory effects by modulation of the immune system, as suggested in previous studies that showed that IL-6 treatment may modulate the hypothalamic-pituitary-adrenal (HPA) axis and autoregulate serum IL-6 levels in response to LPS [21,26,29]. Furthermore, prolonged administration of IL-6 in humans led to adrenal enlargement and identification of IL-6 receptors in the adrenal cortex in mice led to the suggestion that the direct stimulatory effects of IL-6 mediate glucocorticoid release [29]. Increased glucocorticoid secretion leads to the restraining of the further production of pro-inflammatory mediators [29,30], thus preventing propagation of the stress response [29]. We believe that exercise could have induced the release of small doses of IL-6 after each training period in our animals, and this would chronically modulate the HPA axis to stimulate the constant release of glucocorticoids [26,29]. Although we did not measure the serum levels of glucocorticoids, the increase in the expression of glucocorticoid receptors in pulmonary inflammatory cells in the trained animals suggests that modulation of the HPA axis could be one of the anti-inflammatory effects induced by exercise in our model. Taken together, our findings suggest that pulmonary release of IL-6 and IL-10 is a major mechanism involved in the protective effect of exercise in this LPS-induced ALI model.

We observed an interaction effect of LPS and exercise in increasing the levels of IL-1β in BALF, indicating that exercise exacerbates the effect of LPS in increasing IL-1β levels. IL-1β is involved in the repair of the alveolar epithelium in the early phase of ARDS through activation of the epithelial epidermal growth factor/transforming growth factor α pathway [31-33]. One of the most important factors that determine the severity of lung injury in ARDS is the magnitude of injury to the alveolar epithelial barrier. The possibility of decreasing the inflammatory process at an early stage or accelerating tissue repair can be a major determinant of recovery [31]. Although the process of alveolar epithelial repair involves several growth factors, cytokines and various extracellular matrix components, it is possible that a further protective effect of exercise on ALI is to promote early tissue repair through IL1-β release.

Levels of TNF-α were not elevated in BALF after LPS instillation but increased expression of TNF-α in lung tissue was observed in LPS-treated animals. Aerobic exercise resulted in a significant effect on decreasing LPS-induced TNF-α expression. Moreover, values of TNF-α in lung tissue were correlated with the density of neutrophils. TNF-α is a major mediator of sepsis and endotoxin-induced ALI; after endotoxin infusion in animals, the level of TNF-α peaks in one to two hours in the serum and in eight hours in BALF. However, due to its short half-life, TNF-α is rapidly cleared and returns to baseline levels within a few hours [34,35]. We believe that this dynamic of the release and clearance of TNF-α could explain the observed low levels of this cytokine in the BALF of our animals, which was collected 24 hours after LPS instillation. We also observed a striking decrease of eNO in our exercised animals. Elevated NO levels are thought to play a central role in tissue damage observed during septic shock [21]. Previous results suggest that NO release after LPS challenge is mediated through IFN-γ and TNF-α [36]. Based on previous observations that pretreatment with IL-6 decreased the levels of TNF-α, NO and other pro-inflammatory cytokines in a model of endotoxemia in mice [21], we suggest that in our model, the low levels of eNO and TNF-α in the exercised animals could, at least in part, be secondary to the continuous release of IL-6 induced by exercise. The negative correlation between the levels of TNF-α and IL-6 in lung tissue in our animals is compatible with this hypothesis.

The aerobic exercise did not alter the effects of LPS on KC release and protein leakage in BALF. These findings were unexpected because KC is a major neutrophil chemotactic factor, and exercise induced a significant reduction in neutrophil density. KC levels were not measured in plasma or pulmonary tissue, limiting the interpretation of our results.

We observed an effect of exercise on decreasing pulmonary resistance and elastance in LPS-instilled animals. Alteration of resistive forces and tissue viscoelastic properties is a major characteristic of ALI and is associated with alveolar barrier disruption, alveolar edema, surfactant dysfunction, collapse and alveolar inflammation [8]. In our animals, there was no significant effect of exercise on protein leakage, and the decrease of pulmonary resistance and elastance in LPS-instilled exercised mice is likely related to the decrease in pulmonary inflammation. The significant correlation between the levels of eNO and values of resistance and elastance is consistent with this mechanism.

To investigate the role of oxidative stress, we analyzed the enzymatic activities of GST, CAT, GPX, and GR, the levels of MDA and the expression of SOD-2 in lung tissue. The levels of MDA, which were used as an index of oxidative stress, were not different between the LPS group and controls. Previous studies have shown that the levels of MDA peak four to five hours after LPS instillation [37,38]. Changes in MDA levels can be undetectable after 8 or 24 hours [38], as observed in our animals. Moreover, we observed that LPS instillation induced activation of the antioxidant glutathione pathway by increasing the activities of GPX and GR, indicating a possible physiologic response to an oxidative stress [39]. Exercise determined an increase in the expression of SOD-2 in lung tissue. SOD-2 is exclusively localized to the mitochondria, a critical cell organelle both in cellular energy metabolism and cell survival [40]. Overall, SOD-2 is most highly expressed in alveolar type II epithelial cells and alveolar macrophages, cell types with relatively high metabolic capacity, and plays a major role in protecting lung tissue against free radicals [40].

## Conclusions

In conclusion, our results show that aerobic exercise plays an important role in protecting the lung from the inflammatory effects of LPS-induced ALI. The effects of exercise are mainly mediated by the increased expression of anti-inflammatory cytokines and antioxidants, suggesting that aerobic exercise can modulate the inflammatory-anti-inflammatory and the oxidative-antioxidative balance in the early phase of ARDS.

## Key Messages

• Aerobic exercise performed during five weeks prior to LPS instillation attenuates the pulmonary changes induced by LPS.

• The beneficial effects of exercise in this ALI model are mainly mediated by the anti-inflammatory cytokines IL-6 and IL-10 and by the expression of SOD-2.

• Our data suggest that exercise can modulate the inflammatory-anti-inflammatory and the oxidative-antioxidative balance in the early phase of ALI.

## Abbreviations

ALI: acute lung injury; ARDS: acute respiratory distress syndrome; BALF: bronchoalveolar lavage fluid; BSA: bovine serum albumin; CAT: catalase; CTR: control; ELISA: enzyme-linked immunosorbent assay; eNO: exhaled nitric oxide; Ers: respiratory elastance; Exe: exercise; GPX: glutathione peroxidase; GR: glutathione reductase; Gre: glucocorticoid receptors; GSSG: oxidized glutathione; GST: glutathione S-transferase; H & E: hematoxylin and eosin; HPA: hypothalamic-pituitary-adrenal; HPLC: high performance liquid chromatography; IFN: interferon; IL: interleukin; LPS: *Escherichia coli *lipopolysaccharide; MDA: malondialdehyde; PBS: phosphate-buffered saline; PMN: polymorphonuclear; Ptr: tracheal pressure; Rrs: respiratory system resistance; SOD: superoxide dismutase; SPSS: Statistical Package for the Social Sciences; TNF: tumor necrosis factor; V': airflow; V: lung volume.

## Competing interests

The authors declare that they have no competing interests.

## Authors' contributions

CTRG participated in the design of the study, carried out the experimental protocol, the immunohistochemistry reactions and the morphometric analyses, performed the statistical analysis and drafted the manuscript. CGRG participated in the design of the study and helped to carry out the experimental protocol. FMA and FDTQSL helped carry out the respiratory mechanics, the BALF analysis and the morphometric analysis. ACCSD, FAS and TM performed the antioxidants assays and helped draft the manuscript. LFFS performed the histological analysis and the immunohistochemistry quality control, helped with the statistical analysis and was involved in drafting the manuscript. HCCFN contributed to analysis and interpretation of data and was involved in revising the manuscript. RPV participated in the design of the study, carried out the immunohistochemistry reactions and the morphometric analyses. MD conceived the study, performed the histological analysis, performed the immunohistochemistry quality control, performed the statistical analysis and drafted and revised the manuscript. All authors read and approved the final version of the manuscript.

## Supplementary Material

Additional file 1 **Treadmill for mice exercise**. The picture illustrates the treadmill used for mice exercise. Click here for file

Additional file 2 **Treadmill for mice exercise**. The picture illustrates the mice during exercise. Click here for file

Additional file 3 **Video of treadmill with mice**. The video illustrates the mice during exercise. Click here for file
