# Supplementary figures and images for: Protective effects of aerobic exercise on acute lung injury induced by LPS in mice
Source: Crit Care. 2012 Oct 18;16(5):R199. doi: 10.1186/cc11807 (PMC3682301; doi:10.1186/cc11807)

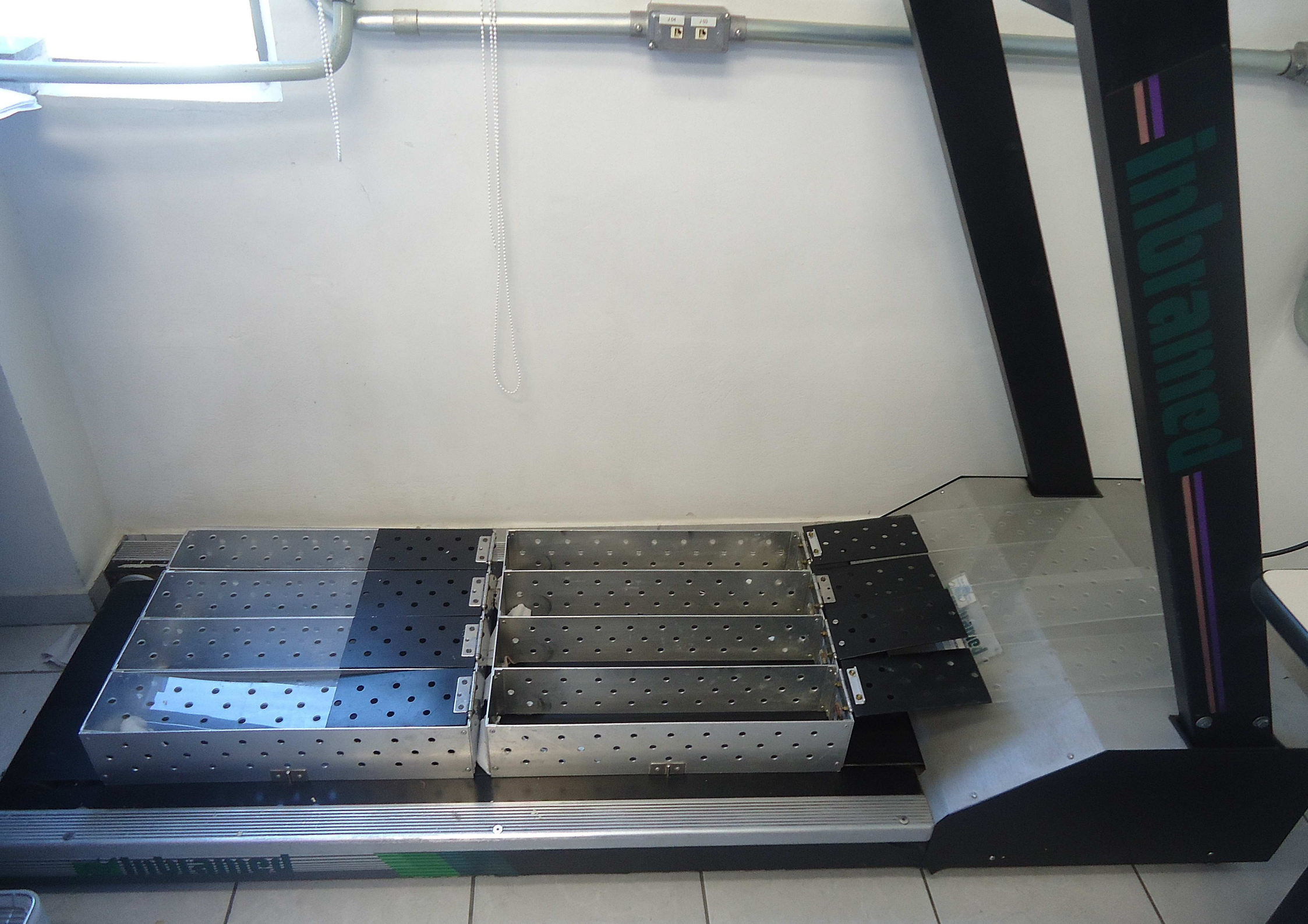

Supplement: Additional file 1 — Treadmill for mice exercise. The picture illustrates the treadmill used for mice exercise. [file cc11807-S1.PDF]

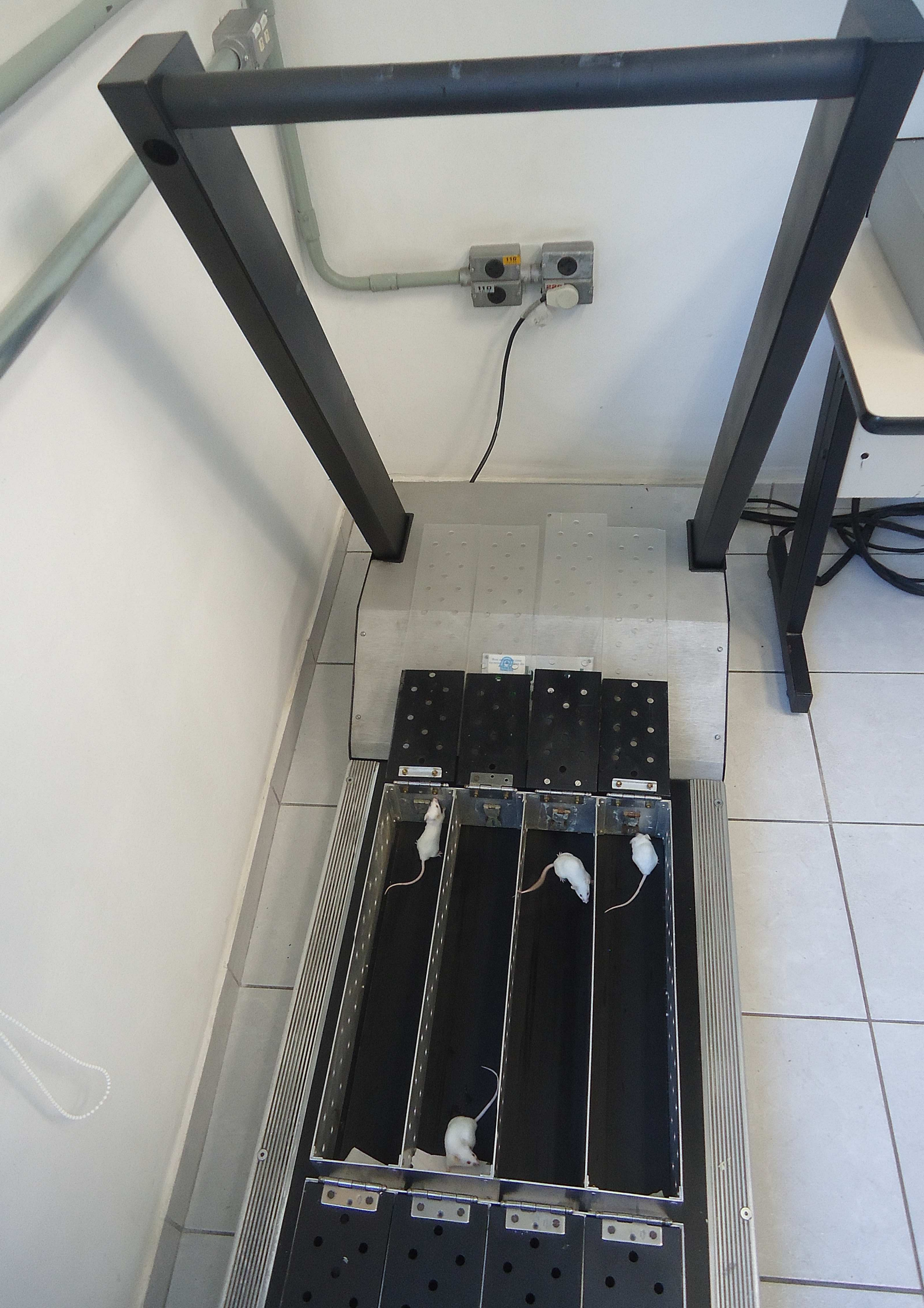

Supplement: Additional file 2 — Treadmill for mice exercise. The picture illustrates the mice during exercise. [file cc11807-S2.PDF]
